# Spin and charge interactions between nanographene host and ferrocene

**DOI:** 10.3762/bjoc.20.89

**Published:** 2024-05-02

**Authors:** Akira Suzuki, Yuya Miyake, Ryoga Shibata, Kazuyuki Takai

**Affiliations:** 1 Graduated School of Science and Engineering, Hosei University, Tokyo 184-8584, Japan; 2 Department of Chemical Science and Technology, Hosei University, Tokyo 184-8584, Japan

**Keywords:** charge transfer, ferrocene, graphene, host–guest, spin interaction

## Abstract

Ferrocene (FeCp_2_) was introduced as a non-magnetic guest molecule to activated carbon fibers (ACFs) as a nanographene-based host having localized spins originating from zigzag edges of graphene. The introduction of the guest molecule was confirmed by FTIR for ACFs-FeCp_2_ introduced at 55 (150) °C (FeCp_2_-ACFs-55(150)). The appearance of satellite Fe_2p_ peaks and the increase in shake-up peak intensity of the C_1s_ in the XPS spectrum proved the emergence of charge-transfer host–guest interaction in FeCp_2_-ACFs-150, supported by the red-shift of the G-band in the Raman spectrum. The six-times enhancement in the spin concentration in FeCp_2_-ACFs-150 compared with ACFs indicates the spin magnetism of the non-magnetic guest FeCp_2_^+^ molecule induced by a charge-transfer host–guest interaction in the nanographene host. The larger ESR linewidth than that expected from the dipolar interaction estimated by the localized spin concentration suggests the exchange interaction between the nanographene and FeCp_2_ spins. The narrowing of the ESR linewidth of FeCp_2_-ACFs-55 upon higher excitation microwave power suggests the inhomogeneity of the environment for FeCp_2_^+^ molecules in the nanographene host. The observed induction of spin magnetism by the interfacial interactions between the nanographene host and the guest molecules will be a promising strategy for developing a new class of molecular magnets.

## Introduction

Nanocarbon host material, which is based on elements free from resource depletion, is attracting much attention due to its potential for creating a new class of functional materials with various guest molecules [1]. In particular, nanosized graphene called nanographene, the macroscopic limit of polycyclic aromatic hydrocarbon molecules, is a magnetic host material with spins localized at edges [2]. The presence of edges greatly modifies the electronic structure of nanographene, which strongly depends on the geometry of the edges [3–5]. Edges at the periphery of nanographene sheets consist of two kinds of geometry: zigzag edges and armchair edges. The presence of the zigzag part in the arbitrarily shaped edges results in the emergence of radical π-electron states called “edge states”, which are spatially localized at the edge site. The edge states appear at the Dirac point at which two linear conduction (anti-bonding) π*- and valence (bonding) π-bands touch each other in the electronic energy bands of graphene. Since the Fermi level is located at the Dirac point for neutral nanographene, edge states are half-filled like singly occupied molecular orbitals (SOMO) of radical states. Namely, nanographene sheets become magnetic and chemically active due to the edge states with localized spins of unpaired electrons [6]. Thus, it is interesting to introduce a magnetic guest molecule into a magnetic nanographene host regarding the development of a new class of magnetic materials.

Oxygen [7–11], nitrogen monoxide molecules [12–13], and potassium clusters having unpaired spins [14–15] have been introduced to nanographene hosts as magnetic guest molecules so far. However, the decomposition of molecules, the vanishment of guest magnetism, etc., after accommodation by the host material prevent magnetic interactions between the host and guest in these systems. The material design should be important in this viewpoint, especially in choosing appropriate guest molecules. Since π electrons extend to in-plane directions in nanographene, a guest molecule with an aromatic ring is promising for significant interaction with the nanographene host through π–π stacking.

Ferrocene (FeCp_2_) is a “sandwich” compound where the two cyclopentadienyl (Cp or C_5_H_5_-) rings sit above and below the Fe^2+^ ion [16]. The electronic structure of FeCp_2_ satisfies the 18-electron rule, so this compound is stable due to a closed L-shell structure in view of the atomic orbitals of Fe and it is a diamagnetic molecule (*S* = 0, no spin magnetism) compared with other metallocenes [17]. However, FeCp_2_ is easily oxidized to a monovalent cation, the electronic structure of which is magnetic (*S* = 1/2). Electron spin resonance (ESR) spectroscopy revealed the spin magnetism of cationic FeCp_2_ accommodated in mesoporous silica (MCM-41) [18]. So, ferrocene is expected to exhibit strong host–guest interactions with a nanographene host through π–π stacking.

Regarding ferrocene as a guest molecule for nanocarbon hosts, carbon nanotubes (CNTs) have been used to accommodate guest ferrocene molecules, where the amount of the charge transfer from ferrocene to CNTs was estimated from the shift of peaks for van Hove singularities in the valence-band photoemission spectrum [19–20]. The magnetic properties of ferrocene encapsulated into CNTs have also been investigated by superconducting quantum interference devices (SQUID) [21–22] and X-ray magnetic circular dichroism (XMCD) spectroscopy [23]. However, only a tiny paramagnetic behavior of encapsulated ferrocene was observed, and no magnetic host–guest interactions were reported due to the diamagnetic nature of CNTs.

Activated carbon fibers (ACFs) consist of a three-dimensional disordered network of nanographite domains, each of which is a loose stack of 3–4 nanographene sheets with a mean in-plane size of 2–3 nm. ACFs have huge specific surface areas (about 2000 m^2^/g [24–25]) due to the presence of nanopores of ca. 1 nm in diameter between the nanographite domains, where various guest chemical species can be accommodated [2]. Thus, ACFs are widely used as nanographene host materials. Interestingly, a ferromagnetic behavior below 120 K was once mentioned for FeCp_2_-adsorbed ACFs, even though no data was shown in the report [26]. It is necessary to clarify the magnetic interactions between the nanographene host and FeCp_2_ guest molecules to achieve a ferromagnet using nanographene host–guest systems.

In this study, we introduced ferrocene to ACFs and investigated the magnetic interaction between the host ACFs and ferrocene as magnetic guest molecule using X-ray photoelectron spectroscopy (XPS), Raman spectroscopy, Fourier-transform infrared (FTIR) spectroscopy, magnetic susceptibility, and electron-spin resonance (ESR).

## Experimental

Commercially available ACFs (Kuraray, FR-20), of which the precursor was a phenol-resin, were pre-heat-treated in a glass tube at 200 °C for 24 hours under 2 × 10^−4^ Pa before the introduction of FeCp_2_ in order to eliminate ambient gas molecules adsorbed in ACFs. The introduction of FeCp_2_ was carried out by exposing ACFs to the vapor phase of FeCp_2_ in the evacuated glass without exposing samples to air after the pre-heat-treatment at temperatures 55 °C and 150 °C (FeCp_2_-ACFs-55, FeCp_2_-ACFs-150), for 18 to 24 hours. The vapor pressure of ferrocene corresponding to each temperature was previously reported (15 Pa for 55 °C, 5.7 × 10^3^ Pa for 150 °C) [27]. In the case of introduction at 150 °C, excessive FeCp_2_ precipitated as crystals on the surface of ACFs, which were removed by heating the FeCp_2_-treated ACFs at 150 °C for 3 hours without FeCp_2_ vapor.

XPS spectra were recorded using a PHI-5600 (ULVAC-PHI) with an Al Kα X-ray source (1486.7 eV) for samples mounted on an indium film. Raman spectroscopy measurements were performed by LabRAM HR Evolution instruments (Horiba) with an excitation laser operated at 532 nm in the wavenumber range from 1000 to 2000 cm^−1^. FTIR spectra were obtained using an FT/IR-6600 (JASCO) in ATR method with a diamond prism. Magnetic susceptibility measurements were carried out by a superconducting quantum interference device (SQUID) magnetometer (Quantum Design, MPMS-XL) in the field of 1 T between 2 K and 300 K, where ca. 30 mg of the samples vacuum-sealed in glass tubes (for ACFs and FeCp_2_-ACFs-150), mounted inside a plastic straw (FeCp_2_) were used. The Weiss temperature Θ and the temperature-independent term of the magnetic susceptibility were obtained by least-square fitting the data of the temperature-dependence of the observed susceptibility χ with the following equation based on a model of the summation of the Curie–Weiss localized magnetism and temperature contribution,


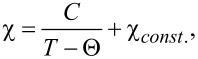


where *C* denotes the Curie constant. The spin concentration *N*_spin_ for each sample was calculated from the Curie constant.

ESR measurements were performed using a conventional ESR X-band spectrometer (JEOL, JES-FA300) at room temperature, where ca 1 mg of samples vacuum-sealed in glass tubes were used. In order to prevent the skin effect, ACFs were ground in a mortar before the measurement.

## Results and Discussion

**Figure 1:**
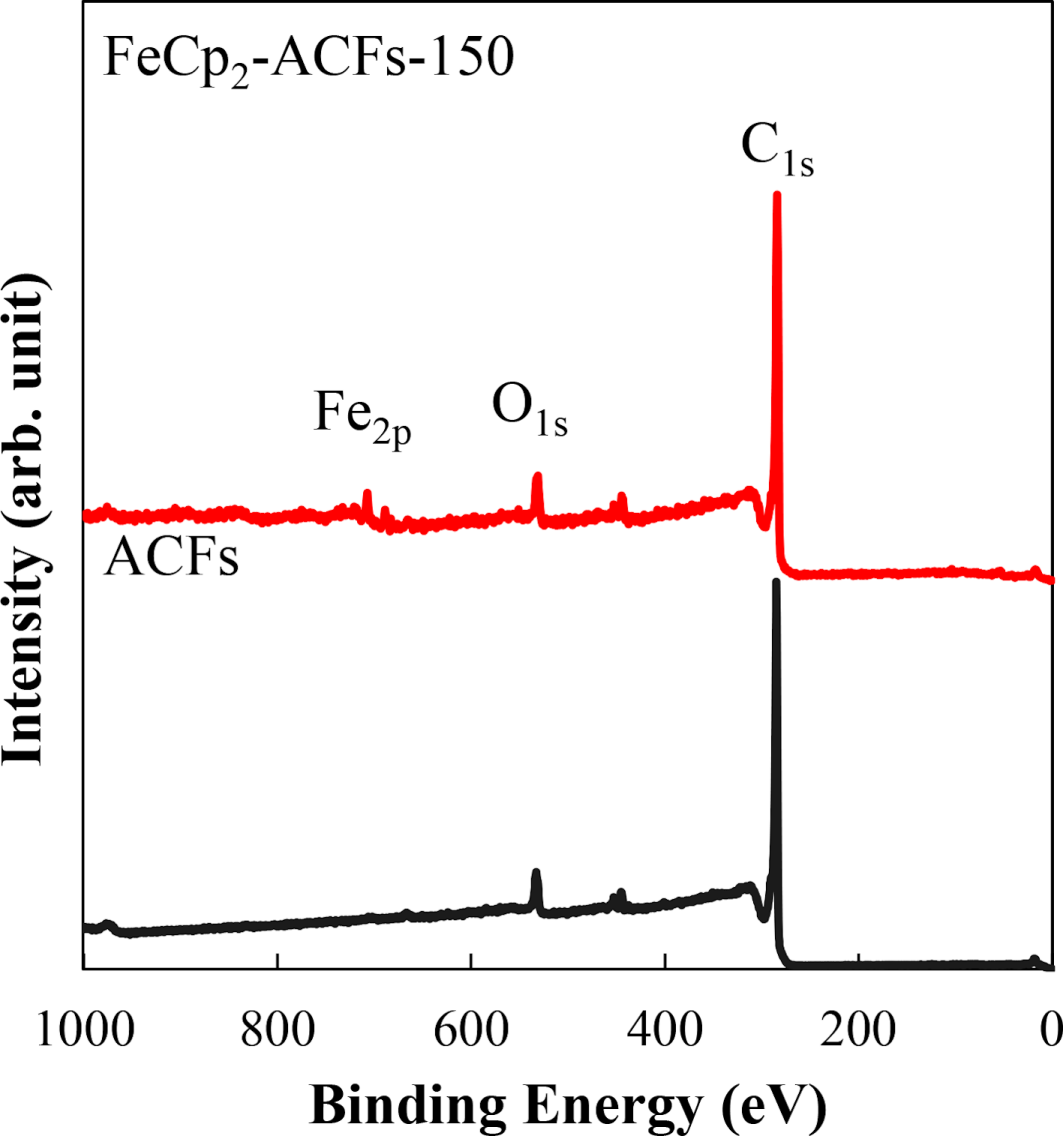
XPS spectra for ACFs and FeCp_2_-ACFs-150. Peaks without labels originate from the indium substrate used for mounting the samples. The base lines of the spectra are shifted vertically from each other for clarification.

XPS spectra acquired in a wide binding energy region for ACFs and FeCp_2_-ACFs-150 are shown in Figure 1. Peaks of C_1s_ and O_1s_ were observed in ACFs, while C_1s_, O_1s_, and Fe_2p_ peaks appeared in the spectrum for FeCp_2_-ACFs-150.

**Figure 2:**
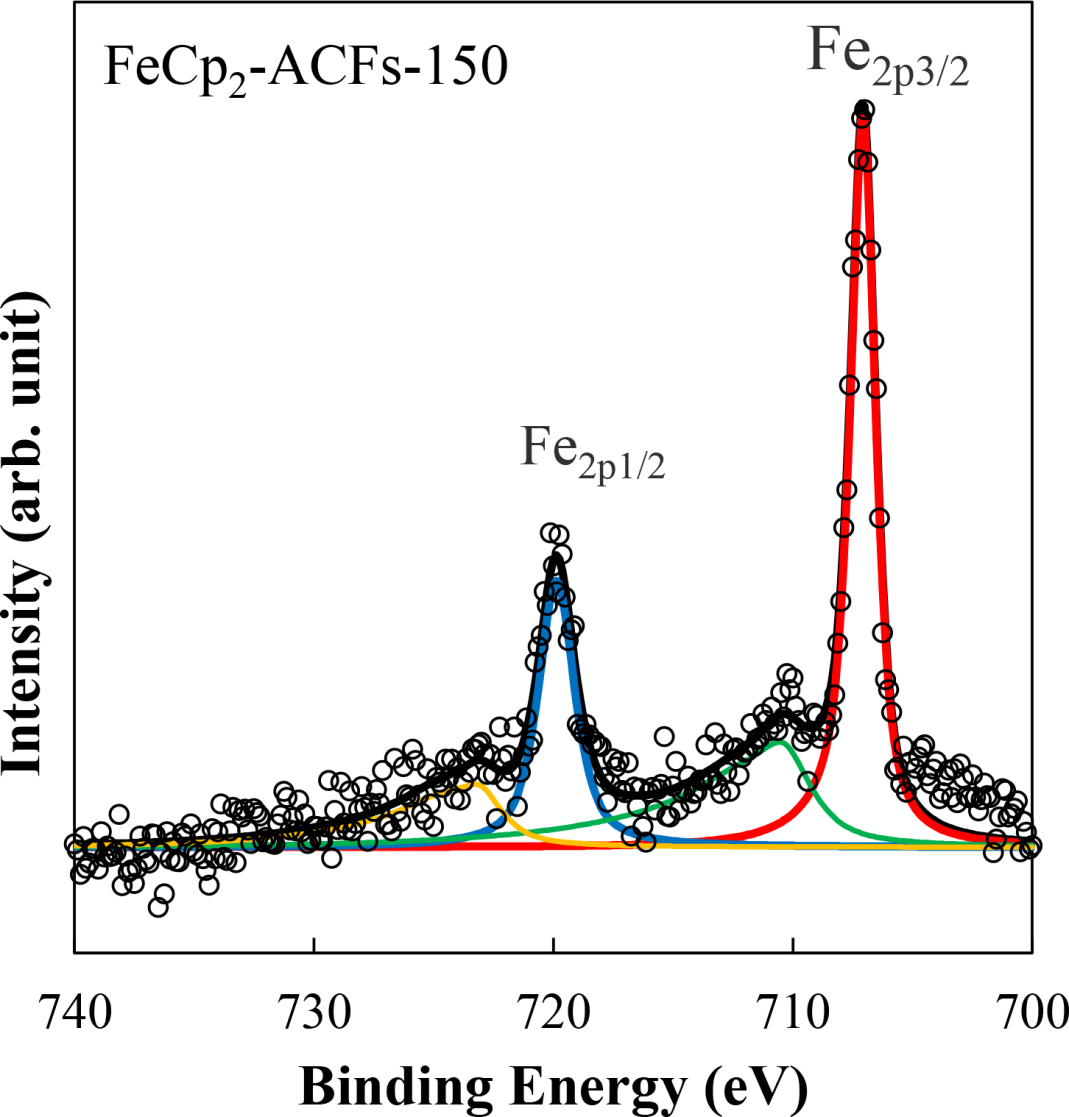
XPS spectrum for FeCp_2_-ACFs-150 in the Fe_2p_ region shown with fitting curves.

Figure 2 shows the Fe_2p_ spectrum for FeCp_2_-ACFs-150 in a narrow binding energy region. The binding energies of the Fe_2p_ peaks are similar to the reported value for FeCp_2_ [16]. So, the Fe_2p_ peaks observed in FeCp_2_-ACFs-150 indicate the successful introduction of the FeCp_2_ molecule into ACFs as nanographene host. In addition to the main peaks, satellite peaks clearly appear at the higher energy side (ca. +3 eV), which indicates that the FeCp_2_ molecules partially become cationized (positively charged) in FeCp_2_-ACFs-150.

**Figure 3:**
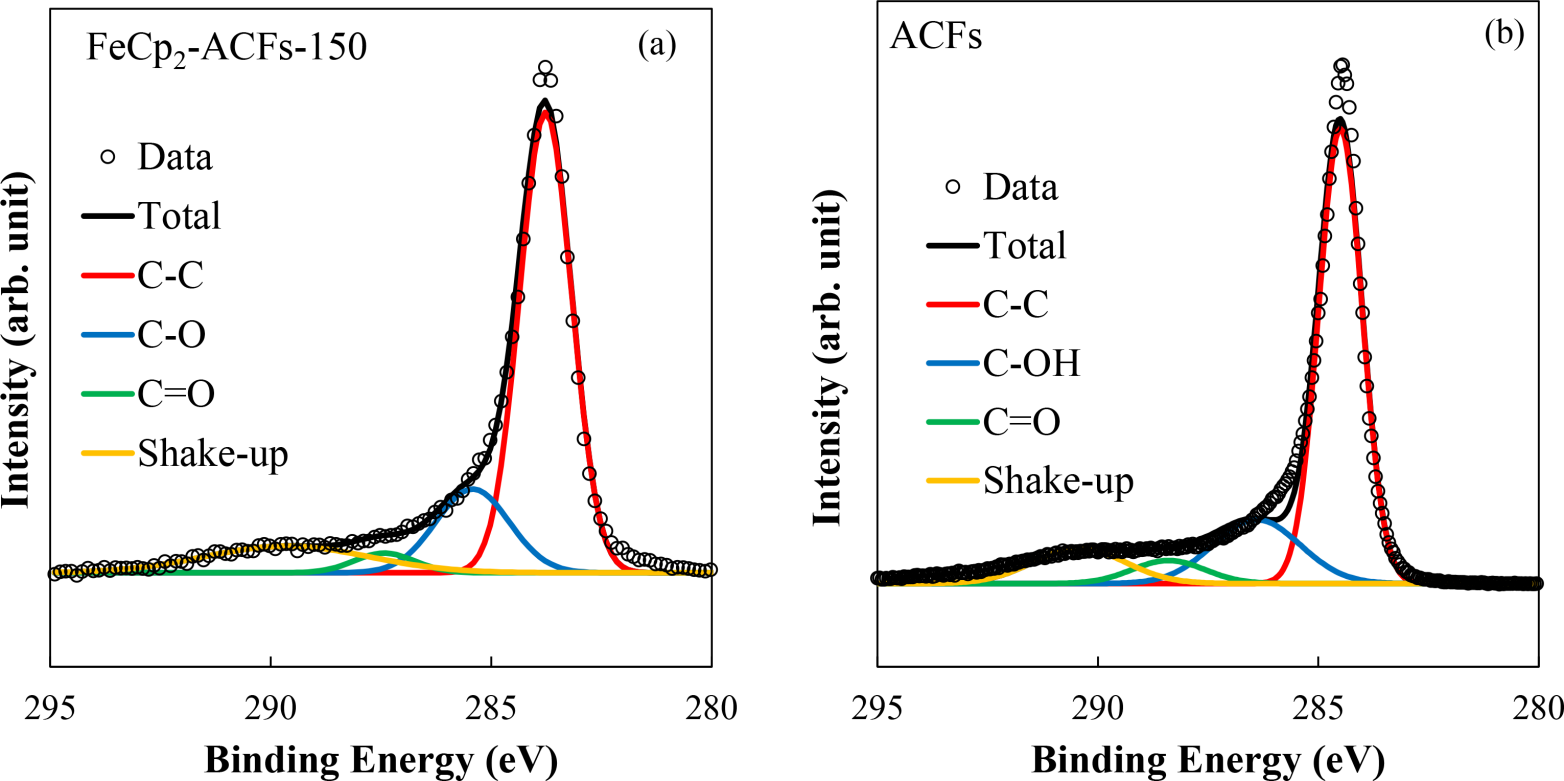
XPS spectra of (left) FeCp_2_-ACFs-150 and (right) ACFs in the C_1s_ region with fitting curves.

Figure 3a and b show the C_1s_ spectra for FeCp_2_-ACFs-150 and ACFs in a narrow binding energy region, respectively.

Table 1 shows peak positions for XPS C_1s_, O_1s_, and Fe_2p_ peaks for ACFs and FeCp_2_-ACFs-150, where elemental abundances are obtained from the peak intensity. The amount of FeCp_2_ is calculated as 0.39 mmol in 1 g of FeCp_2_-ACFs-150 from the total intensity ratio of Fe_2p_. The amount of the cationized FeCp_2_ (FeCp_2_^+^) is obtained as 0.15 mmol/g of FeCp_2_-ACFs-150 according to the intensity ratio of satellite peaks to the main peaks. The O_1s_ peaks mainly come from oxygen-containing functional groups bonded to nanographene because of oxidization by ambient gaseous species. The elemental abundance ratio O/C of 0.07 is the same for ACFs and FeCp_2_-ACFs-150, similar to the carbon atoms ratio at the nanographene's edge part with the in-plane size of 2–3 nm, where the ratio of edge atoms to total carbon atoms is ca. 0.1 with the assumption of a model circular nanographene C_324_H_36_. Thus, oxygen-containing functional groups in ACFs are mainly attached to the edge part of nanographene, being consistent with the higher chemical activity of the edges of graphene [2,6]. Furthermore, the almost same elemental abundance ratio O/C between ACFs and FeCp_2_-ACFs-150 indicates no additional oxidization occurred in the process of FeCp_2_ introduction to ACFs due to contaminated oxygen from ambient gaseous species.

**Table 1:** The peak positions for XPS C_1s_, O_1s_, and Fe_2p_ spectra and abundances of the peak components for FeCp_2_-ACFs-150 and ACFs.

XPS peak	Sample	Binding energy (eV)	Abundance (atom %)

C_1s_ (C=C)	ACFs	284.6	64
	FeCp_2_-ACFs-150	283.7	61
C_1s_ (C-O)	ACFs	286.4	16
	FeCp_2_-ACFs-150	285.4	16
C_1s_ (C=O)	ACFs	288.4	3.8
	FeCp_2_-ACFs-150	287.4	3.0
C_1s_ (shake-up)	ACFs	290.4	9.3
	FeCp_2_-ACFs-150	289.6	12
O_1s_	ACFs	532.9	6.8
	FeCp_2_-ACFs-150	531.4	6.6
N_1s_	ACFs	400.3	0.4
	FeCp_2_-ACFs-150	–	–
Fe_2p_	ACFs	–	–
	FeCp_2_-ACFs-150	707.1, 719.9 710.5, 723.2	0.8 0.5

Peaks of C_1s_ are assigned to sp^2^ carbon atoms (C=C) of nanographene sheets, carbon atoms in/near oxygen-containing functional groups bounded to edges of nanographene sheets (C–O, C=O), shake-up peak by π–π* transition of conduction π electrons (Shake-up) [28]. A more considerable contribution of the plasmon peak in C_1s_ indicates an increase in π-electron carriers for FeCp_2_-ACFs-150. Indeed, the shift of the C=C peak of FeCp_2_-ACFs-150 to the lower energy side indicates an increment of screening effect on photoemission hole by increasing in conduction electrons. Increasing in conduction π electron of nanographene in FeCp_2_-ACFs-150 suggests the charge transfer from FeCp_2_ to ACFs. In this connection, the observed partial ionization of FeCp_2_ is well understood by the structure of ACFs. Nanopores between nanographene domains provide huge spaces for the adsorption of guest molecules inside ACFs [2,6], where only a part of introduced molecules directly face the nanographene with the interfacial host–guest interactions, and the rest is accommodated into the nanopores without significant influences by nanographene domains.

**Figure 4:**
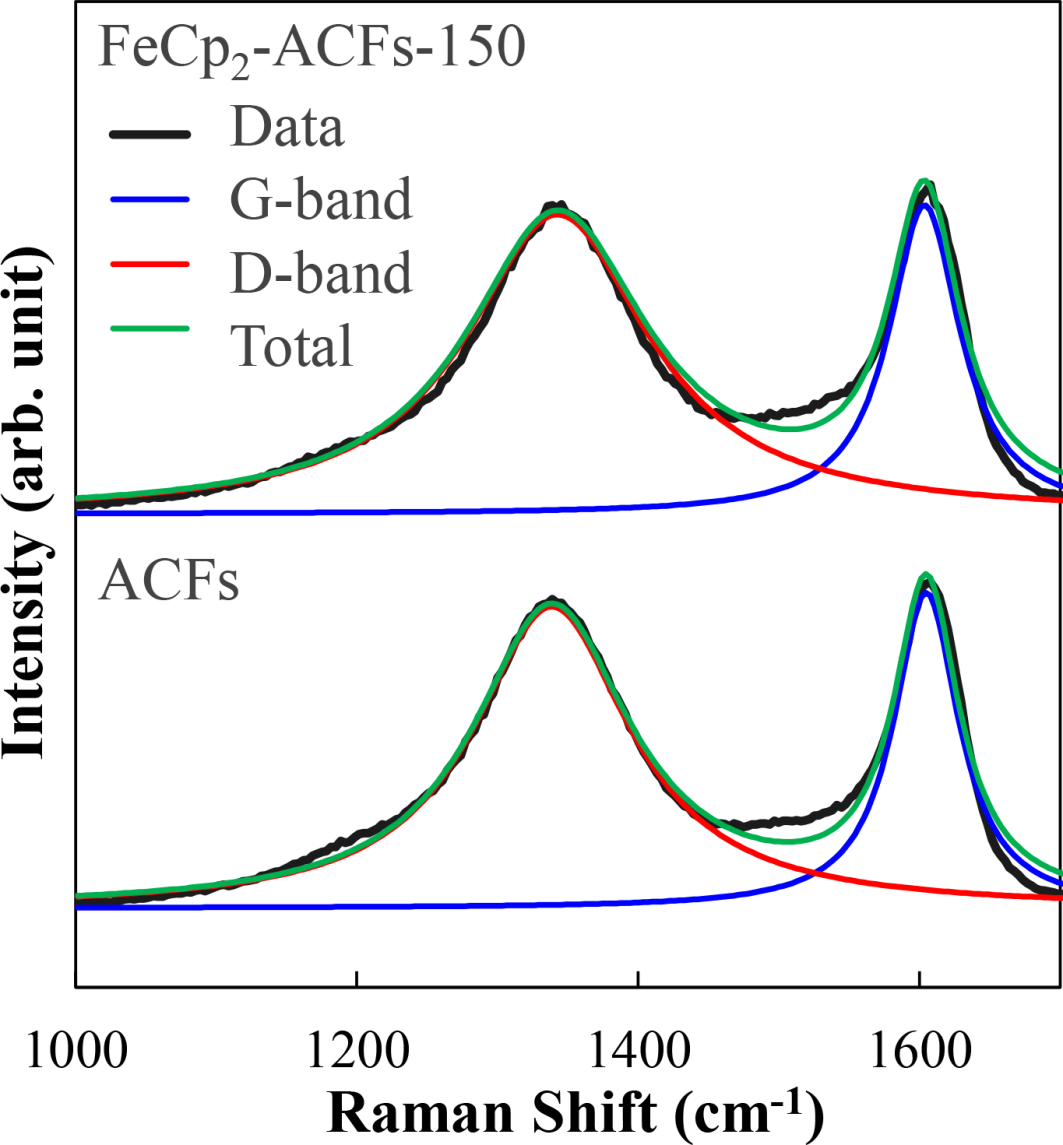
Raman spectra for ACFs and FeCp_2_-ACFs-150. Each raw data (black) was fitted to the G-band (blue) and D-band (red) components, resulting in the total curve (green). The base lines of the spectra are shifted vertically from each other for clarify.

The Raman spectra for both ACFs and FeCp_2_–ACFs-150 shown in Figure 4 exhibit two broad peaks near 1350 and 1600 cm^−1^. The peak around 1600 cm^−1^ corresponds to the Raman-allowed *E*_2g_ mode (G-band) in graphene. The D-band peak around 1350 cm^−1^ is forbidden in ideal graphene crystals but becomes Raman-active by an electron-scattering process due to impurities and edges in crystallites [29]. The G and D-bands were fitted with two Lorentzian curves, as shown in Figure 4. Although characteristic peaks of FeCp_2_ molecules around 1100 cm^−1^ are not obtained in the spectrum for FeCp_2_-ACFs-150 due to their tiny abundance, the G-band for FeCp_2_-ACFs-150 shifts by 3 cm^−1^ to the lower wavenumber side compared to ACFs. The red shift indicates the weakening of C=C bonding in nanographene caused by filling anti-bonding states (π* states) due to electron injection into nanographene. This is consistent with the increment of shake-up peak for C_1s_ in XPS. The Raman D-band also supports charge transfer from FeCp_2_ to nanographene in FeCp_2_-ACFs-150. The intensity ratio of the D-peak to G-peak *I*_D_/*I*_G_ increases from 2.3 for ACFs to 2.4 for FeCp_2_-ACFs-150. The larger *I*_D_/*I*_G_ corresponds to the more significant carrier scattering by introducing FeCp_2_ as a positively charged impurity caused by charge transfer with nanographene in FeCp_2_-ACFs-150. This is also supported by the increase in the linewidth of the G-band from 28 cm^−1^ (ACFs) to 31 cm^−1^ (FeCp_2_-ACFs-150).

**Figure 5:**
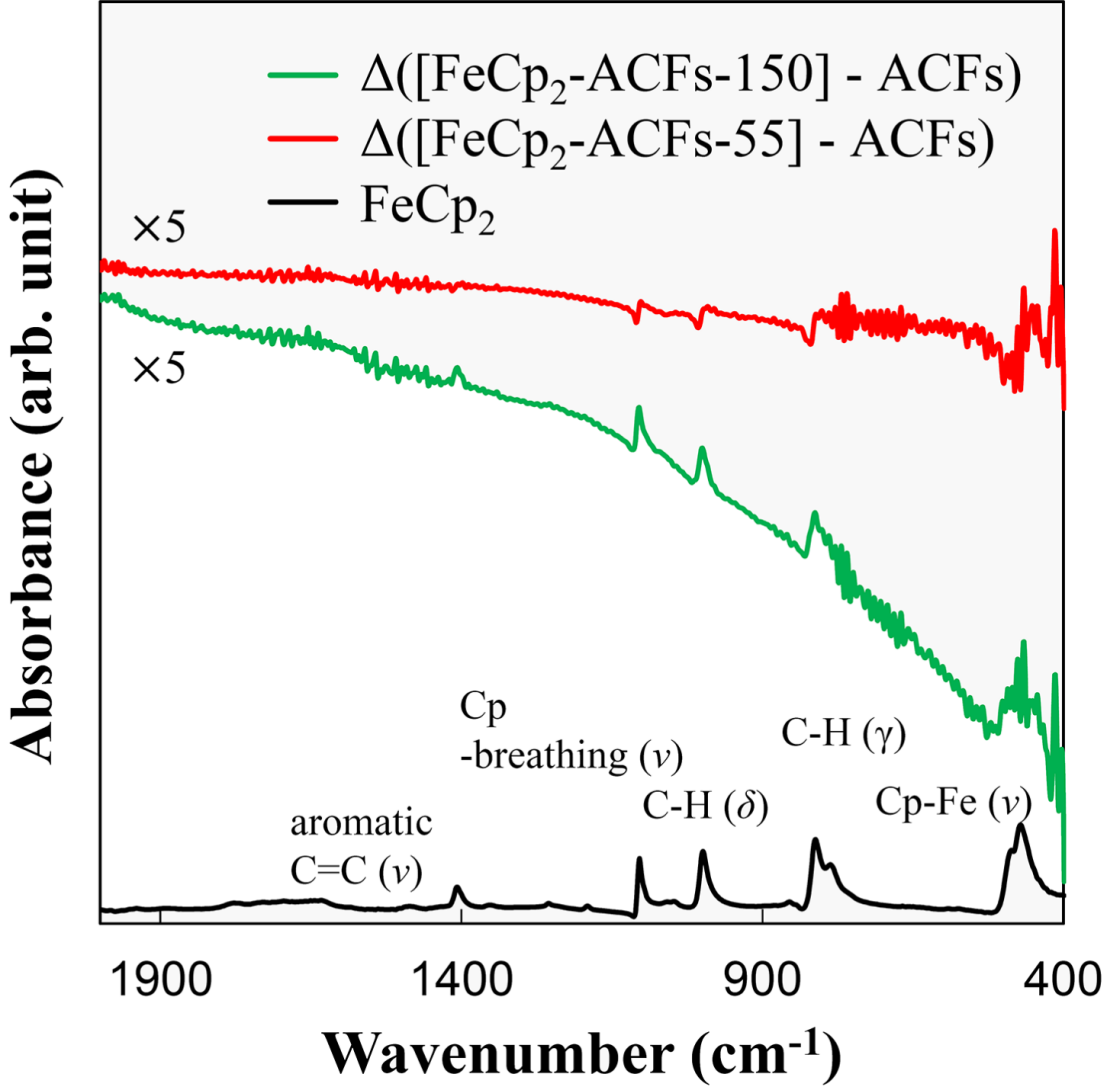
The raw infrared spectrum for FeCp_2_ (black) and differential absorbance spectra for FeCp_2_-ACFs-55 (red) and FeCp_2_-ACFs-150 (green) after subtracting the ACFs spectrum as a background signal. The subtracted spectra are magnified five times, where the base lines are shifted vertically from each other for clarity. Raw spectra for FeCp_2_-ACFs-55 and FeCp_2_-ACFs-150 are shown in Figure S1 in Supporting Information File 1.

Figure 5 shows IR spectra for FeCp_2_, and the infrared spectra differences from that of ACFs for FeCp_2_-ACFs-150 (Δ([FeCp_2_-ACFs-150]-ACFs) and FeCp_2_-ACFs-55 (Δ([FeCp_2_-ACFs-55]-ACFs). The difference spectra exhibit peaks for vibration modes of Cp–Fe (ν), C–C (ν), C–H (γ), C–H (δ), Cp-breathing (ν) typical for FeCp_2_ molecular vibration [30]. These spectra also indicate the successful introduction of FeCp_2_ to ACFs and that most FeCp_2_ maintains its molecular structure inside the nanographene host in both of FeCp_2_-ACFs-55 and FeCp_2_-ACFs-150. Moreover, the higher peak intensities of FeCp_2_ molecular vibrations in the spectrum for FeCp_2_-ACFs-150 than FeCp_2_-ACFs-55 suggests that more guest molecules are introduced in FeCp_2_-ACFs-150. This is quite reasonable, taking the much higher vapor pressure of FeCp_2_ (5.7 × 10^3^ Pa) into account in the process of guest molecular adsorption into ACFs for FeCp_2_-ACFs-150 than FeCp_2_-ACFs-55 (15 Pa). Here, it should be noted that the vibrational spectra are more distorted due to electromagnetic shielding effects by the conductive nature of graphene-based materials upon IR excitation. Thus, the “apparent” negative absorption peak in the spectrum of FeCp_2_-ACFs-55 is caused by the phase shift of the IR electromagnetic wave by shielding effects of the conductive nanographene assembly. Interestingly, the FeCp_2_ molecular vibrational peaks appear as typical positive peaks for FeCp_2_-ACFs-150, indicating that most molecules are present inside the nanopores of ACFs without significant interactions such as charge transfer and electromagnetic shielding. This is well consistent with the observed partial cationization of the guest molecules for FeCp_2_-ACFs-150 in XPS due to nanopore structure of the nanographene network in ACFs.

The host–guest interaction between guest FeCp_2_ and host ACFs is most pronounced in the magnetic susceptibility measurements. The magnetic susceptibility for each sample shows the Curie–Weiss-type temperature dependence with temperature-independence susceptibility χ_const_, mainly composed of the orbital diamagnetism by core and π electrons. Regarding FeCp_2_-doped ACFs, ferromagnetism has been reported [26], but all of our samples showed only paramagnetism, and no ferromagnetism was observed in the present study.

**Figure 6:**
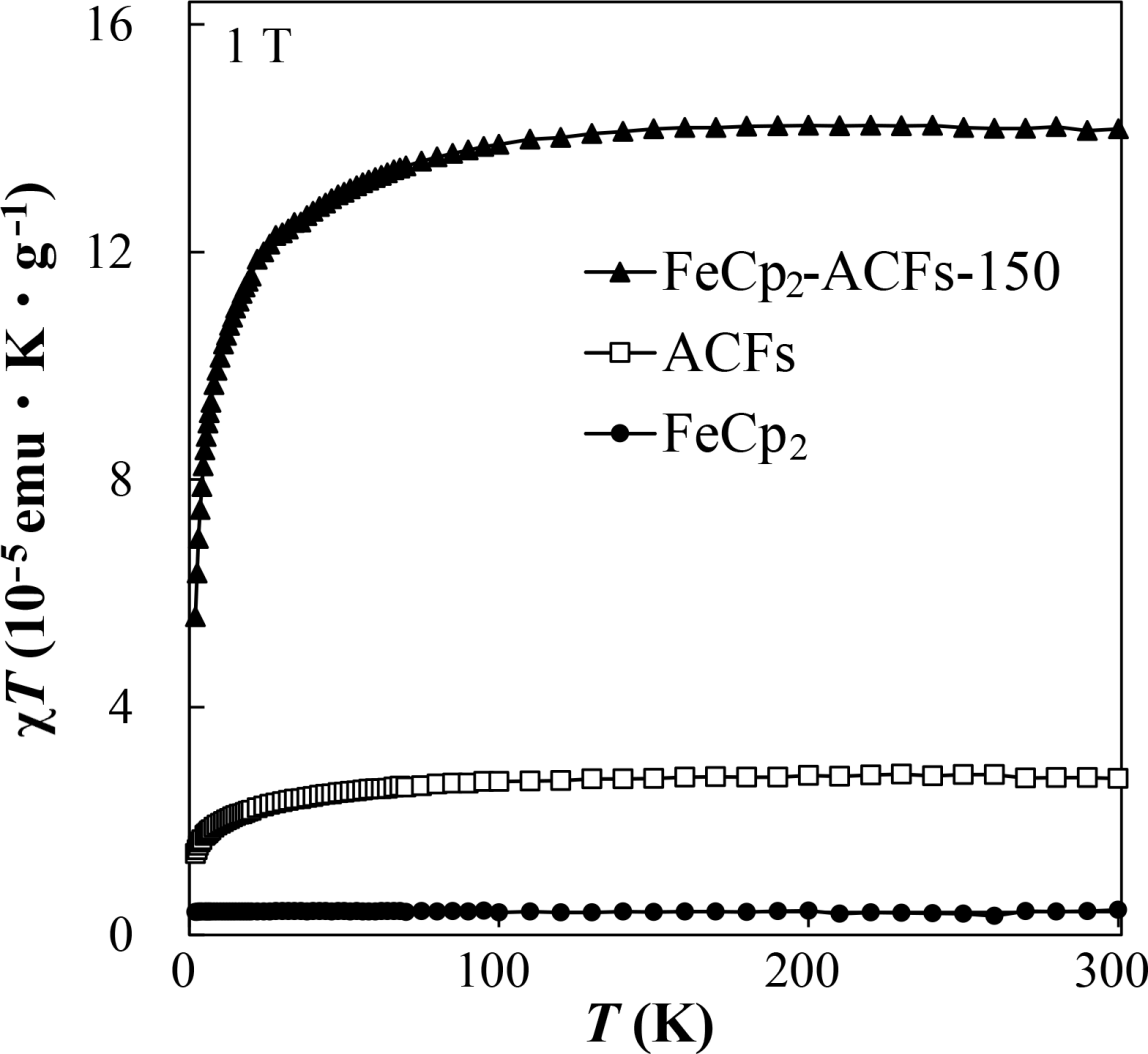
The temperature (*T*) dependence of the magnetic susceptibility χ for FeCp_2_-ACFs-150, ACFs, and FeCp_2_ measured at 1 T, where the vertical axis denotes the χ multiplied by *T*. The temperature-independent diamagnetic contribution to the magnetic susceptibility is subtracted.

The temperature-independent term of the magnetic susceptibility χ_const_ for ACFs and FeCp_2_-ACFs-150 were obtained as −10 × 10^−6^ and −0.8 × 10^−6^ emu g^−1^, respectively. The reduction in the absolute value of χ_const_ suggests the upshift of the Fermi energy from the Dirac point in the electronic band of nanographene in ACFs, being consistent with charge transfer from FeCp_2_ observed in XPS. The decrease in the absolute value of the Weiss temperature Θ from −6.4 K for ACFs to −0.1 K for ACFs-FeCp_2_-150 indicates the change in the character of the observed spins. The temperature dependences of the magnetic susceptibility χ multiplied by temperature *T* for FeCp_2_-ACFs-150, ACFs, and plain FeCp_2_ are shown in Figure 6, where χ_const_ was subtracted. The quantity χ*T* tells us an estimation for the effective spin concentration modified by spin-exchange interactions at each temperature. The χ*T* for ACFs remains constant in the temperature region above 50 K. However, it becomes decreasing below 10 K as temperature decreases. This is featured as the localized spin paramagnetism with antiferromagnetic interaction. ACFs exhibit the localized spin paramagnetism by edge-states of nanographene, as reported [2]. As expected from the diamagnetic (no spin magnetism) electronic structure of FeCp_2_, plain FeCp_2_ shows only tiny paramagnetism caused by impurities. On the other hand, after FeCp_2_-introduction to ACFs, the χ*T* remarkably increases for FeCp_2_-ACFs-150, as shown in Figure 6. Indeed, *N*_spin_ for FeCp_2_-ACFs-150 is about six times larger than that for non-doped ACFs (0.39 × 10^20^ g^−1^ for ACFs and 2.2 × 10^20^ g^−1^ for FeCp_2_-ACFs-150). The results indicate that FeCp_2_ in FeCp_2_-ACFs-150 becomes cationized (FeCp_2_^+^) and magnetic (*S* = 1/2) by the charge-transfer interaction with nanographene.

Here, we quantitatively discuss the observed spin magnetism induced by charge-transfer interactions between host and guest with the results of XPS. The additional spin concentration by FeCp_2_ introduction into ACFs is 1.8 × 10^20^ g^−1^ for FeCp_2_-ACFs-150, being equivalent to 0.30 mmol for 1 g of FeCp_2_-ACFs-150. The ratio of the satellite peak to the main peak of the XPS Fe_2p_ spectrum tells us 0.15 mmol of FeCp_2_^+^ for 1 g of FeCp_2_-ACFs-150, which is in the same order as the observed spins induced by charge-transfer host–guest interactions. Considering the accuracy of elemental abundance by XPS (≈0.2 atom %), this is enough reasonable coincidence.

The Weiss temperature also supports the emergence of FeCp_2_^+^ spin magnetism. The absolute value of Θ decreases from −6 K to −0.09 K after FeCp_2_ introduction to ACFs. The wavefunction of the edge-state is coupled to each other through the π-electron systems in the nanographene sheet, resulting in antiferromagnetic interactions. In contrast, the wavefunction (molecular orbital) of FeCp_2_^+^ has a more isolated nature, and the exchange interactions between cation spins are less than those for edge-state spins. The apparent reduction in Θ for FeCp_2_-ACFs-150 is attributed to the contribution of FeCp_2_^+^ spins having less exchange interaction in the observed magnetic susceptibility. So, the spin magnetism of the guest molecule is induced by host–guest interactions in the nanographene host.

**Figure 7:**
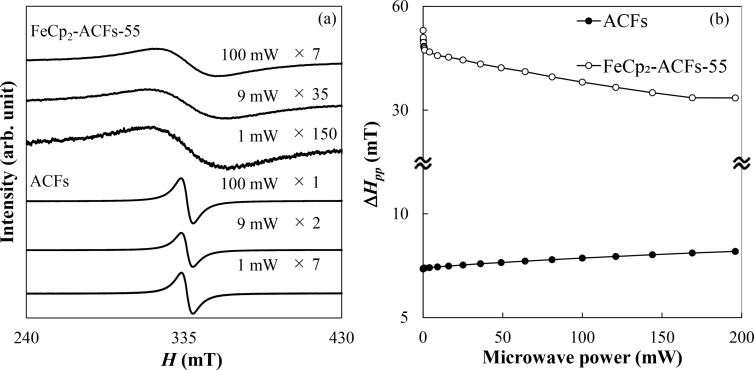
a) ESR spectra of ACFs and FeCp_2_-ACFs-55 with different excitation microwave power. Each spectrum was magnified for clarity, where the base lines are shifted vertically from each other for clarity. b) The excitation microwave power dependence of ESR linewidth (Δ*H*_pp_) for ACFs and FeCp_2_-ACFs-55.

Despite the less interacting nature of FeCp_2_^+^ spins than that of edge-state spins, the ESR measurement proves the presence of the magnetic interaction between spins of the ACFs host and the guest FeCp_2_ molecule. Figure 7a shows the ESR spectra for ACFs and FeCp_2_-ACFs-55 at the excitation microwave powers of 1, 9, and 100 mW. The ESR linewidth of the spectrum for FeCp_2_-ACFs-150 was extremely broad to analyze the spectra, such as estimation of the linewidths and intensities, where the spectra are merged with the baseline contribution on the wider field range, being hard to distinguish from each other (Figure S2 in Supporting Information File 1).

ESR for ACFs and FeCp_2_–ACFs-55 gives a *g*-value of 2.0019 for ACFs and a *g*-value of 2.003 for FeCp_2_-ACFs-55, being in good agreement with the reported value for edge-state spins of nanographene in ACFs (*g* = 2.002) [26]. These *g*-values are almost constant within the error bar in the excitation microwave power-dependence measurement. Figure 7b shows the excitation microwave power dependence of the ESR linewidth Δ*H*_pp_ for ACFs and FeCp_2_-ACFs-55. If we only consider the magnetic dipole interaction, Δ*H*_pp_ is proportional to *N*_spin_ [26], so Δ*H*_pp_ for FeCp_2_-ACFs-150 should be six times larger than that for ACFs, according to the magnetic susceptibility results. However, the ESR of FeCp_2_-ACFs-150 results in a broad linewidth undistinguishable from the baseline (Figure S2 in Supporting Information File 1). Even ACFs-FeCp_2_-55 shown in Figure 7a, where FeCp_2_ was introduced at 1/300 lower pressure gives Δ*H*_pp_ about seven times larger than ACFs in ESR. The observed ”excess” broadening factor for FeCp_2_-ACFs-150 and FeCp_2_-ACFs-55 in ESR is attributed to the exchange interaction between spins. Generally, the exchange interaction between identical spins results in the narrowing of the ESR peak (exchange narrowing). However, the exchange between non-identical spins broadens the ESR spectrum. In FeCp_2_-ACFs-55 and FeCp_2_-ACFs-150, the exchange interaction between nanographene spin and FeCp_2_^+^ spin (non-identical spins) contributes in addition to the magnetic dipolar interaction.

**Figure 8:**
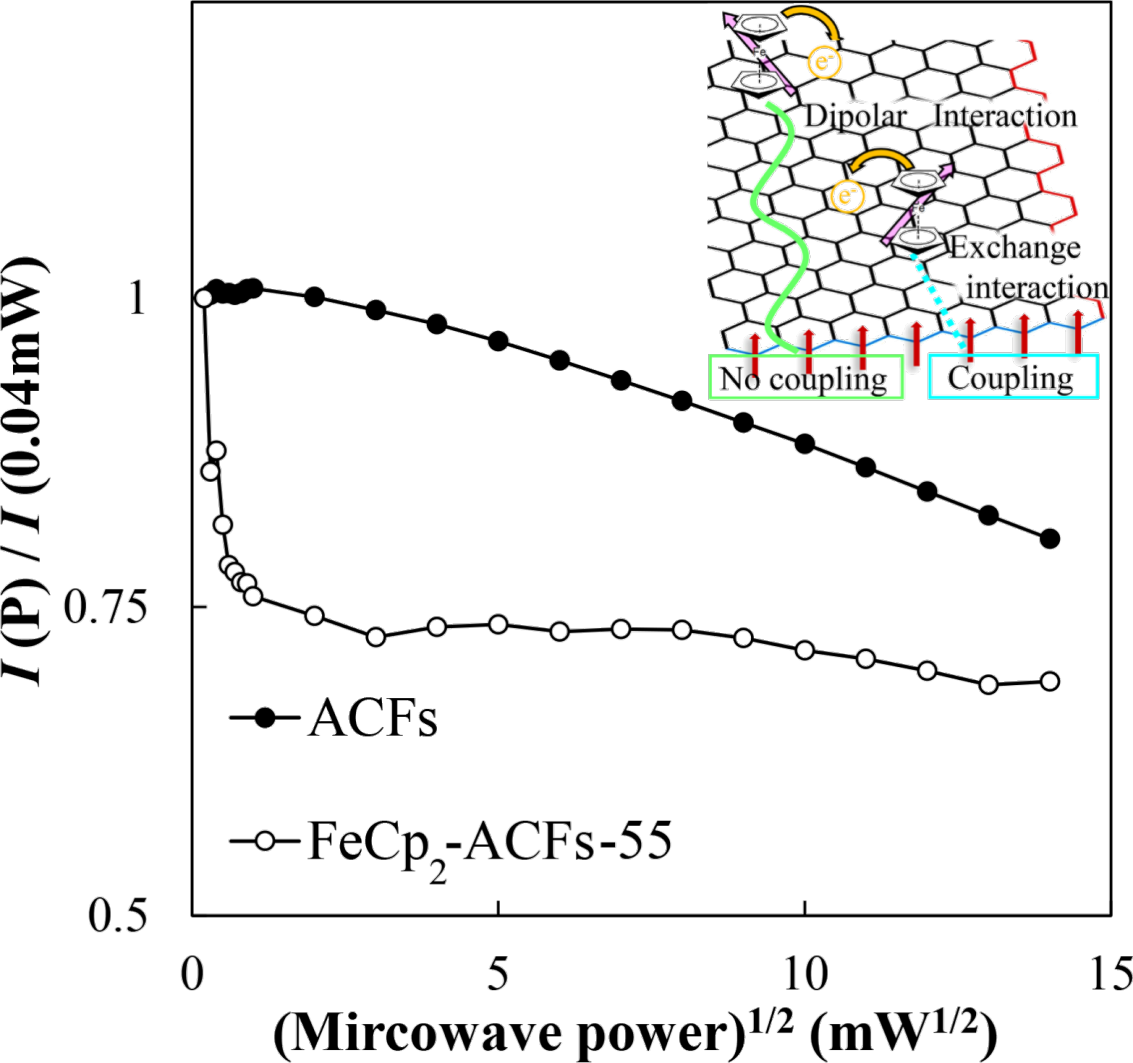
The square root of the excitation microwave power dependence of the relative ESR intensities for ACFs and FeCp_2_-ACFs-55. Each intensity is normalized by that at microwave power of 0.04 mW. The insert is an illustrative sketch to explain spin and charge interactions between the nanographene host and FeCp_2_ guest molecules.

Figure 8 shows the square root of excitation microwave power dependence of relative intensities for ACFs and FeCp_2_-ACFs-55. At higher excitation power conditions, the relative intensities of ESR decrease because of a larger excitation rate than the spin relaxation rate (saturation), accompanied by linewidth broadening (saturation broadening). ACFs show moderate saturation phenomena with simple saturation broadening as the excitation power increases, where the coupling with conduction electrons in nanographene sheets is the primary path for spin energy relaxation. In Figure 8, the relative intensity for FeCp_2_-ACFs-55 suddenly decreases in the lower excitation power region. It shows a more saturated nature than ACFs at the same power region despite the larger conduction carrier than ACFs. The more saturating nature for FeCp_2_-ACFs-55 is well explained by the contribution of FeCp_2_^+^ spin having a more isolated nature than edge-state spins, consistent with the magnetic susceptibility results. However, the Δ*H*_pp_ of FeCp_2_-ACFs-55 suddenly decreases even at the lower excitation power similar to the relative intensity and remains a decreasing trend despite its easily saturated nature. These behaviors suggest that spins have an inhomogeneous environment for spin relaxation. In FeCp_2_-ACFs-55, the adsorption site of FeCp_2_ is not unique, and each FeCp_2_ interacts with edge-state spins at the edges and π-electron carriers on nanographene sheets in different manners in ACFs as illustratively shown as the inset of Figure 8.

## Conclusion

Non-magnetic guest molecules with aromatic moiety were successfully introduced into the nanographene host. The charge-transfer interaction with the nanographene host in FeCp_2_-ACFs induces the localized spin magnetism of the guest molecule (cationized FeCp_2_). The presence of the exchange interaction by hybridization between FeCp_2_^+^ orbitals and edge-state orbitals is suggested in addition to the magnetic dipolar interaction. The observed induction and modulation of the spin magnetism by the interfacial interactions between magnetic nanographene host and guest molecules will give insight into a new class of developing methods of molecular magnets.

## Supporting Information

File 1 Supporting figures.

## Data Availability

The data that supports the findings of this study is available from the corresponding author upon reasonable request.
