# Supplementary material for: Spin and charge interactions between nanographene host and ferrocene
Source: Beilstein J Org Chem. 2024 May 2;20:1011–9. doi: 10.3762/bjoc.20.89 (PMC11070967; doi:10.3762/bjoc.20.89)
Supplement: File 1 — Supporting figures. [file Beilstein_J_Org_Chem-20-1011-s001.pdf]

BEILSTEIN JOURNAL OF ORGANIC CHEMISTRY

Supporting Information

for

Spin and charge interactions between nanographene host and ferrocene

Akira Suzuki, Yuya Miyake, Ryoga Shibata and Kazuyuki Takai

Beilstein J. Org. Chem. **2024,** *20,* 1011–1019. doi:10.3762/bjoc.20.89

Supporting figures

License and Terms: This is a supporting information file under the terms of the Creative Commons Attribution License (https://creativecommons.org/licenses/by/4.0). Please note that the reuse, redistribution and reproduction in particular requires that the author(s) and source are credited and that individual graphics may be subject to special legal provisions.
The license is subject to the *Beilstein Journal of Organic Chemistry* terms and conditions: (https://www.beilstein-journals.org/bjoc/terms)

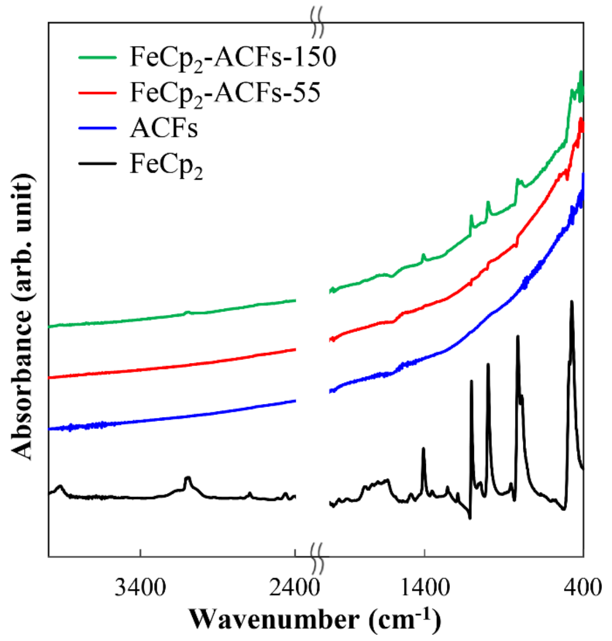

Figure S1: The raw infrared spectra for $FeCp_2$ (black), ACFs (blue), $FeCp_2$-ACFs-55 (red) and $FeCp_2$-ACFs-150 (blue). The region related to N_2 and CO_2 peaks was hidden, and the base lines of the spectra are shifted vertically from each other for clarify.

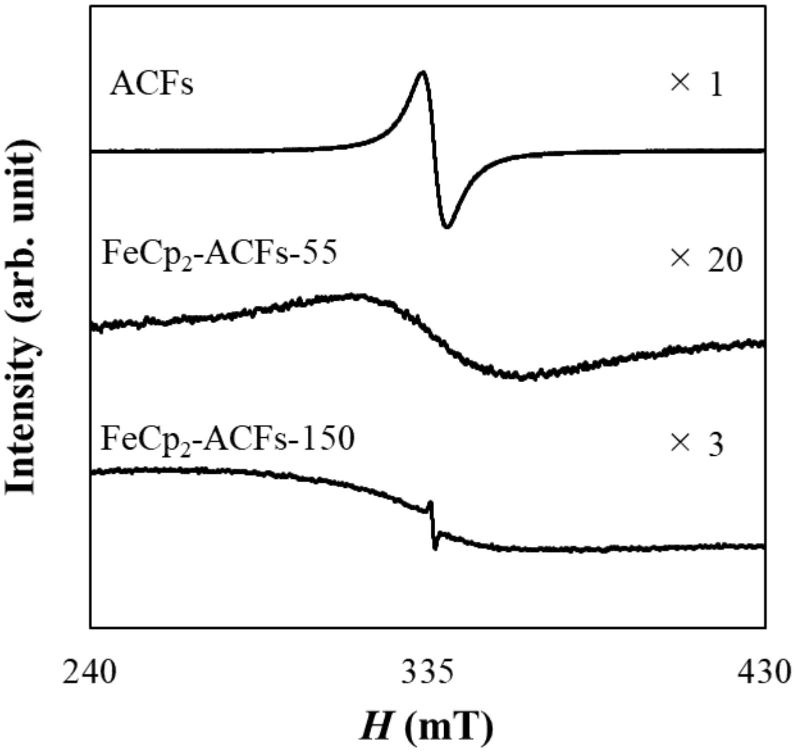

Figure S2: ESR spectra for ACFs, $FeCp_2$-ACFs-55, and $FeCp_2$-ACFs-150 at 1 mW excitation microwave power. The small sharp peak for $FeCp_2$-ACFs-150 comes from the impurities in glassware for measurement. The base lines of the spectra are shifted vertically from each other for clarify.
